# Chemical Composition of Wild Collected and Cultivated Edible Plants (*Sonchus oleraceus* L. and *Sonchus tenerrimus* L.)

**DOI:** 10.3390/plants13020269

**Published:** 2024-01-17

**Authors:** M. Ángeles Botella, Pilar Hellín, Virginia Hernández, Mercedes Dabauza, Antonio Robledo, Alicia Sánchez, José Fenoll, Pilar Flores

**Affiliations:** 1 Departamento de Biología Aplicada, Escuela Politécnica Superior de Orihuela (EPSO), CIAGRO-Universidad Miguel Hernández, 03312 Orihuela, Alicante, Spain; mangeles.botella@umh.es; 2 Instituto Murciano de Investigación y Desarrollo Agrario y Medioambiental (IMIDA), c/Mayor s/n, La Alberca, 30150 Murcia, Murcia, Spain; mariap.hellin@carm.es (P.H.); virginia.hernandez5@carm.es (V.H.); mercedes.dabauza@gmail.com (M.D.); alicia.sanchez15@carm.es (A.S.); jose.fenoll@carm.es (J.F.); 3 ISLAYA Consultoría Ambiental, S.L., c/Ntra. Sra. de Fátima 34, 30151 Santo Ángel, Murcia, Spain; islaya@islaya.es

**Keywords:** neglected wild species, sugars, acids, carotenoids, chlorophylls, vitamin C, total phenolic compound, sensory attributes

## Abstract

The present work investigates the nutritional and bioactive composition, as well as the organoleptic and sensory properties, of *S. oleraceus* and *S. tenerrimus*, two wild plant species traditionally used in the gastronomy of the Mediterranean area. Additionally, the effect of cultivation on leaf composition was assessed to explore their potential for large-scale production and commercialization from the point of view of possible losses or gains in quality. Both species were characterized as a good source of bioactive compounds, such as vitamins, pro-vitamins and carotenoids, with health-promoting and antioxidant properties that are highly appreciated. The sensory profile revealed a good general acceptance of *S. oleraceus* and *S. tenerrimus*, indicating that they could be included in the diet. Although the cultivation of *S. oleraceus* resulted in a decrease in the concentration of phenolic compounds when compared to wild-harvested plants, the opposite occurred for vitamin C. In *S. tenerrimus*, cultivation also increased the concentration of other compounds with important nutritional and healthy properties, such as sugars, organic acids and *β*-carotene. The results of the composition, organoleptic and sensory properties of *S. oleraceus* and *S. tenerrimus* support the idea of their potential to be used as edible leafy vegetables and as promising assets for functional foods.

## 1. Introduction

Nowadays, food is very often based on a limited number of species. Thus, diversification to consume a broad range of plant species could help to improve nutrition and health. Although currently underutilized, wild edible plants are part of the cultural and genetic heritage of different regions of the world. Moreover, in rural areas, around 80% of people consume wild plants as a source of nutrients and primary health care [1]. With regard to the EU, according to the FAO [2], 100 million people consume wild foods and, specifically, the contribution of wild greens to the Mediterranean diet has been widely documented [3]. Wild plants can be a good source of nutrients such as vitamins, proteins, minerals, and dietary fibres [4,5,6]. In addition to their nutritional potential, wild plants have been reported to possess therapeutic activity as natural antidiabetic agents [7], as well as antibacterial and antitumour activities [8,9]. Furthermore, there is interest in finding food products, which can be not only a source of bioactive compounds, but also food sources for *haute cuisine*, as chefs are increasingly showing interest in plants that have been consumed as part of a traditional background. In this sense, species that have been part of the Mediterranean diet can be an alluring genetic resource to obtain food products. Łuczaj and Pieroni [10] reported the use of wild food plants by different *avant-garde* restaurants around the world, which included a very varied selection of local wild plants in their offering.

Within the underutilized edible plants, the genus *Sonchus* includes annual, biennial, and perennial herbaceous species [11] distributed in Europe, Asia, and Africa. Several *Sonchus* species have been used as food in poor areas of the world [12,13]. The edible part of both *S. oleraceus* and *S. tenerrimus* is composed of the basal leaves and tender raw stems, which are used in salads or stews. Some authors indicate that, within wild plants, *Sonchus* species present a higher potential nutritional and medicinal quality than any other leafy vegetable [14]. *Sonchus* species are particularly rich in vitamin C, but also contain vitamin A, vitamin K and multiple B-group vitamins and minerals (Ca, K, Mg and P), as well as dietary fibre [15]. They also contain a range of antioxidants, including flavonoids, phenolic compounds and carotenoids [16,17]. Therefore, extracts of different *Sonchus* species have been found to possess antioxidant, anti-inflammatory, antimicrobial and anticancer activities [18,19,20]. Specifically, works in the literature indicate that *Sonchus oleraceus* has pharmaceutical properties against bacterial infection [8,21], HIV [22], inflammation [23], diabetes [24], tumours [9], diarrhoea and enteritis [25], anxiety [26] and aging [27]. Recently, a diet including hemp seed and *S. oleraceus* has proven to expand the lifespan of aged mice; therefore, this seems to be a promising and sustainable strategy that could improve human health [28]. In view of this evidence, species such as *S. oleraceus* and *S. tenerrimus*, which have been traditionally consumed in the Mediterranean areas, may be of interest not only because of their culinary applications, but also due to their potential benefits for human health as a good source of bioactive compounds, which are found in greater concentrations than those of some cultivated vegetables such as spinach and cabbage [15].

To meet the demand of regional and international markets, wild edible plants are largely collected from wild populations [2]. This type of harvesting has certain advantages over cultivation: it is cheaper, no pesticides are used, it encourages the protection and maintenance of wild populations and their habitats, etc. However, there are also important disadvantages, such as the lack of homogeneity and continuity of supply and even the risk of extinction of ecotypes and species due to uncontrolled harvesting. On the other hand, cultivation can help to alleviate wild-gathering pressure, ensure a continuous supply of raw material and correct botanical identification or achieve a greater standardization of genotypes and a greater control over quality maintenance [2]. Thus, exploring the potential for the cultivation of edible wild plants of interest would contribute to the promotion of their use as an alternative and sustainable source of leafy green vegetables with nutritional, medicinal and culinary applications.

Despite the different uses reported in the Mediterranean region and other areas [3,29,30], information about the organoleptic and sensory characteristics of *S. oleraceus* and *S. tenerrimus* is scarce. Moreover, no information is available on the adaptation of these species to cultivation and their effect on the composition of the edible part of the plant. The aim of the present work was to reinforce the previous existing information on the nutritional composition of *S. oleraceus* and *S. tenerrimus* [14], studying the profiles of soluble sugars, organic acids, carotenoids, chlorophylls, vitamin C and total phenolic compounds, as well as their organoleptic and sensory properties; in addition, this study evaluated the effect of cultivation on leaf composition to explore the potential of the large-scale production of these plant species for commercialization, from the point of view of possible quality losses or gains.

## 2. Results and Discussion

The study focused on *S. oleraceus* and *S. tenerrimus* species from the *Asteraceae* family on the basis of previous ethnobotanical, biochemical and medical studies [3,6,10,15,18], considering that they were local (autochthonous) plants or had adapted to regional conditions, that they possessed a well-known traditional use (high number of use reports in the Iberian Peninsula) [29], that their palatability was ensured and that they had high concentrations of healthy compounds.

### 2.1. Germination

On the whole, the germination rate of wild species is lower than that of commonly cultivated species [31]. Germination rate was found to be very different when determined in Petri dishes in the growth chamber and in the field. In Petri dishes, germination rate, calculated as the percentage of germinated seeds after 15 days, was relatively high for both species, with values of 75.8 ± 6.2% for *S. oleraceus* and 82.1 ± 8.0% for *S. tenerrimus*. Germination results in the field were lower than those obtained in Petri dishes, and much lower for *S. tenerrimus* (17.8 ± 11.2%) than for *S. oleraceus* (49.9 ± 8.0%). Considering the results, and in order to achieve the maximum homogeneity in the cultivation trial, it was decided to germinate the seeds initially in Petri dishes, and transfer them afterwards to seedbeds during the autumn–winter cycle.

### 2.2. Nutritional Value

In this section, the results of primary and secondary metabolite content in cultivated *S. oleraceus* and *S. tenerrimus* are presented. The soluble sugars detected were glucose, fructose and sucrose (Table 1). Whereas glucose concentration was similar in both species, sucrose was significantly higher (3.7 times) in *S. tenerrimus* than in *S. oleraceus*, and fructose concentration, which showed the lowest values of all of the individual sugars, was 35% higher in *S. tenerrimus* than in *S. oleraceus*. Accordingly, total sugar content was higher in *S. tenerrimus* than in *S. oleraceus*, due to a significantly higher content of sucrose in the first one. Compared to other cultivated leafy vegetables, glucose and fructose content in both species was lower (up to two and thirteen times, respectively) than that observed in lettuce [32,33], but higher (up to three and four times, respectively) than that recorded in supermarket lamb’s lettuce samples [34]. The concentration of sucrose in *Sonchus* was five times (*S*. *oleraceus*) and eighteen times (*S*. *tenerrimus*) higher than that found in baby leaf lettuce [35] and two times (*S*. *oleraceus*) and seven times (*S*. *tenerrimus*) higher than that found in lamb’s lettuce [34], but similar (*S*. *oleraceus*) and three times (*S*. *tenerrimus*) lower than the one observed in lettuce [33]. Sucrose content in leaves constitutes an energy reserve and is correlated with photosynthetic activity and plant growth rate [35]. Therefore, large variations can be expected among different species and growing conditions.

In addition to sugars, organic acids also play an important role in the taste perception of vegetables, as they can modify the taste of sugars. Thus, Fabian and Blum [36] indicated that malic acid enhances the perception of sucrose, whereas citric acid masks the perception of sucrose [37,38] and fructose [39]. Our results revealed a substantially higher concentration of citric, malic and tartaric acids than that of the other organic acids in both *Sonchus* species (Table 1). In other common leafy vegetables, such as lettuce and lamb’s lettuce, citric and malic acids are among the most concentrated organic acids. While lettuce is also high in fumaric acid [33], lamb’s lettuce is rich in quinic acid [34]. Contrary to what happened in soluble sugars, both *Sonchus* species showed higher levels (by two times) of total organic acids than lettuce [32]. Although no differences were found between both *Sonchus* species regarding the content of citric, succinic, quinic, isocitric and glutamic acids, some differences in the organic acid profile were observed; significantly higher concentrations of malic, ketoglutaric and shikimic acids were found in *S. tenerrimus* than in *S. oleraceus*, while tartaric, fumaric and malonic acids were significantly more concentrated in *S. oleraceus* than in *S. tenerrimus*.

Chlorophyll concentrations were similar in both species (Table 1) and, according to our data, the total chlorophyll concentration was up 13 times lower than that reported for spinach [40] and other cultivated leafy vegetables, such as chicory, dandelion, garden rocket and wild rocket [41], but two times higher than in lamb’s lettuce [33]. The role of chlorophylls as bioactive compounds has been less studied than that of other compounds, such as carotenoids or phenols, but they are known to be among the bioactive compounds with a high antioxidant activity [42,43]. Therefore, chlorophylls may play a role in human health [44], providing benefits due to their antioxidant activity, anti-inflammatory and anticancer properties [40] and antimutagenic properties [45].

Although carotenoids have been widely investigated as bioactive compounds, studies on the composition of *Sonchus* species are very scarce, with more references for *S. oleraceus* than for *S. tenerrimus*. Total carotenoid content was similar for both species (88 and 99 µg g^−1^ for *S. oleraceus* and *S. tenerrimus*, respectively). For *S. oleraceus*, works in the literature indicate both slightly lower (53 µg g^−1^) [17] and higher (140 µg g^−1^) [46] values than ours. For *S. tenerrimus*, the study by Guil-Guerrero et al. [14] reported slightly lower (57 µg g^−1^) total carotenoid content than our results for this species.

Carotenes accounted for 56% and 58% of the total carotenoid content in *S. oleraceus* and *S. tenerrimus*, respectively, and, as in a high number of plant species, *all*-*trans*-*β*-carotene was the major carotene [47], accounting for 49% (*S. oleraceus*) and 50% (*S. tenerrimus*) of total carotenoids (Table 1). *Cis* isomers 9-*cis*-*β*-carotene and 13-*cis*-*β*-carotene were also detected in both species, although in a much lower proportion than the *all*-*trans* isomer. It is known that green leafy vegetables are a rich source of *β*-carotene [48], which, together with *α*-carotene and *β*-cryptoxanthin, constitute the main provitamin A carotenoids in the human diet [49]. Moreover, *β*-carotene acts by quenching free radicals and thus attenuating oxidative stress, preventing the progression of eye diseases [50]. When compared with other studies in *S. oleraceus*, the values of *β*-carotene concentration obtained in our experiment were lower [51], similar [17] and higher [46] than those previously reported (63, 49 and 28 µg g^−1^, respectively). The discrepancies found in the literature can be attributed to natural genetic differences between individuals of the same species and to the fact that the profile of carotenoids and other metabolites is strongly influenced by the environment and agricultural management [52]. With regard to other cultivated species, our results showed a higher content of *β*-carotene in both *Sonchus* species than in lettuce (18 µg g^−1^), roquette (33 µg g^−1^), chicory (36 µg g^−1^) and cress (36 µg g^−1^) [53], but lower values than for dandelion (63 µg g^−1^), garden rocket (80 µg g^−1^) and wild rocket (70 µg g^−1^) [41].

As for xanthophyll, our results confirm what had been widely reported, i.e., that the predominant xanthophyll in vegetables is lutein [54,55]. In our study, it represented 23% (*S. oleraceus*) and 19% (*S. tenerrimus*) of the total carotenoids. Similarly, in previous works with *S. oleraceus*, lutein accounted for about 25% of total carotenoids [46]. In different types of lettuce [33] and in lamb’s lettuce [56], lutein was also the major xanthophyll, showing similar values to those obtained for both *Sonchus* species. In other cultivated leafy vegetables, such as kale, spinach, chicory, dandelion, garden rocket and wild rocket, lutein was also the major xanthophyll, but it was present at a higher concentration [41,57]. The role of lutein in human health is based on its photoprotective and antioxidant function to prevent or delay the macular degeneration that occurs with age, as well as cataract formation [58,59]. Moreover, it plays a role in protection against various chronic diseases [60]. Violaxanthin was the second most abundant xanthophyll for both *Sonchus* species and showed concentrations in the range of those previously reported for *S. oleraceus* [46] and other commonly consumed leafy vegetables [41]. In agreement with previous studies, the concentration of neoxanthin was lower than that of violaxanthin [61], and values were in the range of other green leafy vegetables [41]. As with other carotenoids, neoxanthin and violaxanthin are predicted to have potential as general antioxidants [62]. In spite of representing a low percentage of xanthophyll cycle pigments, zeaxanthin has previously been detected in species belonging to the *Asteraceae* family (*C. vesicaria, S. asper* and *S. oleraceus*) and other leafy vegetables [46,63]. However, it was not detected in our *Sonchus* samples. In contrast, to our knowledge luteoxanthin has not been previously reported in *Sonchus* species, but relatively high concentrations, representing 9.9% and 11.3% of total xanthophylls, were found in *S. oleraceus* and *S. tenerrimus*, respectively. Due to its structural characteristics, this xanthophyll has shown potential anti-*Helicobacter pylori* [64] and anticancer [65] activities. More recently, luteoxanthin has shown an inhibitory activity of the human ACE-2 (angiotensin-converting enzyme) receptor that facilitates the entry of SARS-CoV-2, thus being considered a potential candidate for the development of specific therapeutic drugs against COVID-19 [66].

In the review performed by Li and Yang [15], the authors point to vitamin C as the most studied nutritional component of the *Sonchus* species, reporting amounts from 250 to 779 mg kg^−1^, a range in which the concentrations found in our study are included. As previously reported, vitamin C content was significantly higher in *S. oleraceus* than in *S. tenerrimus* [14] (Table 1), with values that would supply the average daily recommended amounts for women (75 mg) if at least 142 g (*S. oleraceus*) or 172 g (*S. tenerrimus*) were consumed. Compared to lettuce, the use of *Sonchus* in the diet can provide 2–3 times more vitamin C concentration [32,33]. Other leafy vegetables such as lamb’s lettuce contained lower or higher vitamin C than both *Sonchus* species, depending on the nutritional treatment received [56], which highlights the impact of growing conditions on the final nutritional composition of vegetables.

With regard to the content of total phenolic compounds, results showed a significantly higher (1.7 times) concentration in *S. tenerrimus* than in *S. oleraceus* (Table 1). Although values were lower than those found in the literature [25], probably due to the aqueous extraction carried out in the present study, results showed that the consumption of *Sonchus* in the diet can provide a 3–5 times higher total phenols content than other commonly consumed leafy vegetables such as lettuce [32].

### 2.3. Sensory Attributes

**Figure 1:**
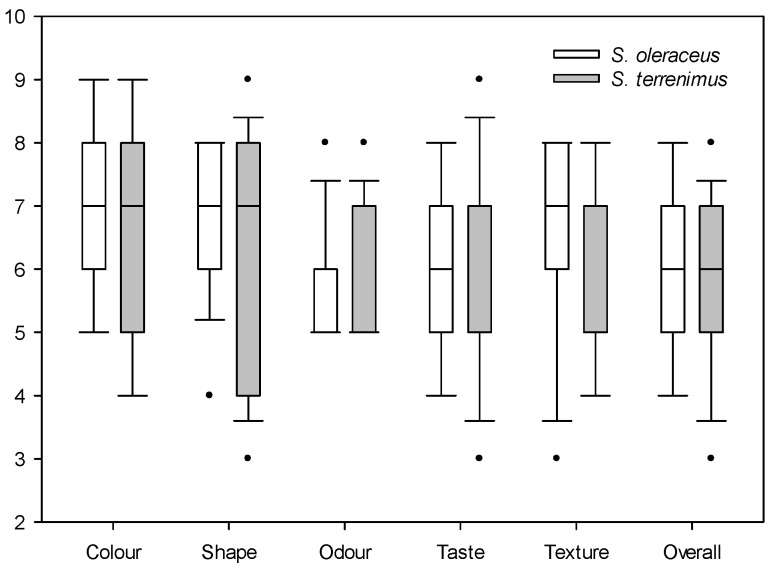
Box plot of the qualitative satisfaction evaluation of cultivated *S. oleraceus* L. and *S. tenerrimus* L. Dots indicate outliers.

**Figure 2:**
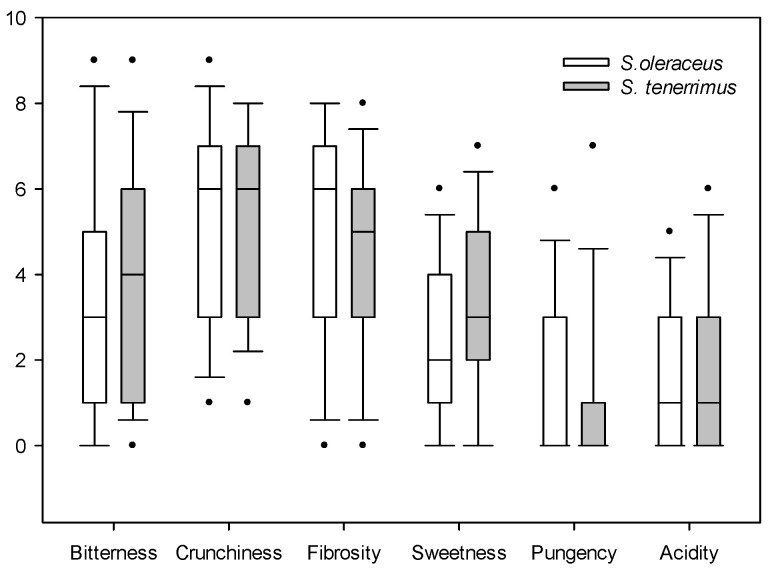
Box plot of the organoleptic descriptive tests of *S. oleraceus* L. and *S. tenerrimus* L. Dots indicate outliers.

For sensory evaluation, the median of each of the evaluated parameters is presented: when data are asymmetric, the median is more useful because the mean will be distorted by outliers (Figure 1 and Figure 2). Both *Sonchus* species obtained similar ratings in the evaluation of satisfaction with colour, shape, odour, taste and texture, with a relatively high general acceptance (overall appreciation), since the mean values were six to seven out of a total of nine (Figure 1). The satisfaction value of visual attributes such as colour and shape was relatively high and similar for both *Sonchus* species. Odour was the attribute that presented the lowest value, with a median of five for both species. Finally, taste and texture were rated similarly by tasters for both species, with acceptable median values. The second valuation that was carried out focused on specific taste characteristics (Figure 2).

No significant differences in bitterness, crunchiness, fibrosity, sweetness, pungency and acidity were detected between both species. Bitterness and sweetness showed marked differences between the testers, having low median values, and being slightly superior for *S. tenerrimus* than for *S. oleraceus*. From the consumer’s point of view, both *Sonchus* species were considered relatively crunchy, but the high variability in the evaluation was noteworthy. Fibrosity presented similar values for both species, and they were not considered to be pungent nor acidic. All of these values have been obtained by evaluating raw materials; therefore, when dressed in salads or used as condiments, they may be more appreciated by the consumer. In fact, it is interesting to note that when these plants were dressed with oil and salt, the panel of tasters showed a much better acceptance. However, this was not the subject of the present study and these data are not presented here.

### 2.4. Wild-Gathered vs. Cultivated Plants

**Figure 3:**
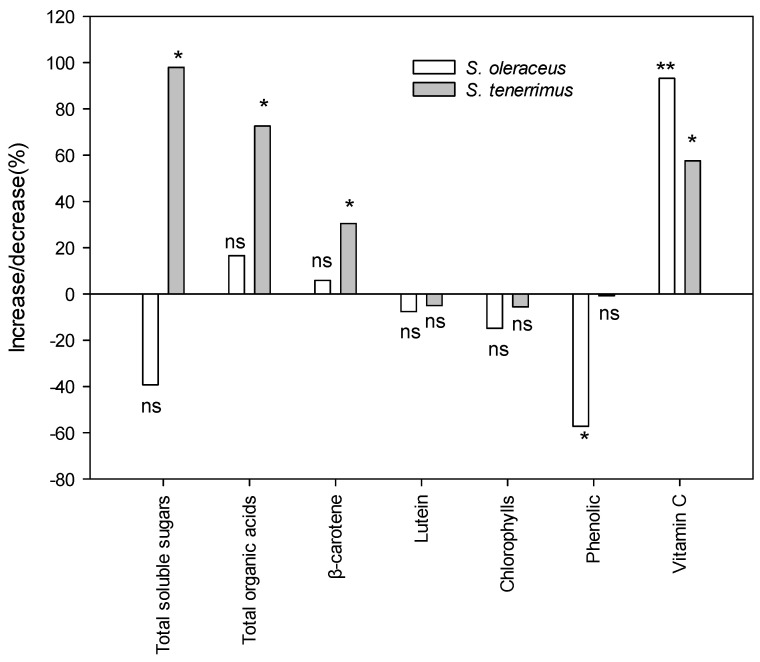
Increase/decrease percentage of metabolite content in cultivated plants with respect to wild plants. Bars are mean values. For each bar, * and ** are significant differences between cultivated and wild relatives at the 5 and 1 levels of probability, according to Kruskal–Wallis’ test; n.s., non-significant at *p* = 5%.

Cultivated plants are often considered qualitatively inferior to wild-gathered plants, and this refers not only to the content of metabolites related to flavour, but also to the content of bioactive compounds. Figure 3 shows the changes in the content of the different metabolites related to the organoleptic and functional quality of the studied species when cultivated and wild plants were compared. Additionally, primary and secondary metabolites from wild harvested plants are presented in the form of supplementary information (Tables S1 and S2). Total soluble sugars and organic acids increased significantly with cultivation in *S. tenerrimus*. However, in *S. oleraceus* no significant differences were detected between wild and cultivated relatives, probably due to the large variability observed in wild plants from different locations. Although results indicated that cultivation could decrease the concentration of phenolics (as observed for *S. oleraceus*), the opposite occurred with other compounds with important nutritional and health properties such as *β*-carotene in *S. tenerrimus* and vitamin C in both species.

Within each species, growing conditions can have a major impact on plant composition and quality, especially with regard to secondary metabolite content [67]. Previous works studying the changes in bioactive compounds that occur with cultivation have shown both increases and decreases or even no effect of cultivation on the bioactive composition of several wild species [68,69]. The discrepancies found in the literature can be attributed not only to the response specificity of each species, but also to the fact that results depend to a large extent on the growth conditions of the wild-gathered and cultivated plants. In general, differences in composition between wild and cultivated plants, which have been reported in previous studies and also in the present one, could be attributed to various abiotic factors such as light, temperature, irrigation or nutrient level [70,71] and also to biotic factors such as pests and diseases. In particular, when plants grow in the wild, they may be exposed to different adverse situations (drought, nutrient deficiency, poor-quality soils), which leads to the synthesis of protective compounds, especially those secondary metabolites that are synthesized as a plant response to stress, such as phenolic compounds [72]. In contrast, the synthesis or accumulation of other metabolites may be compromised by limiting growth conditions; that is the case with N and its deficiency [56]. Wild *Sonchus* samples were collected in different habitats in the Region of Murcia. Thus, the mean values that were obtained can be considered as representative results of the studied geographical region and its climatic conditions (in general, poor soil and low rainfall). Under these conditions, cultivation caused a decrease in the content of compounds involved in the plant’s defence against stress (phenolic compounds). In contrast, cultivated *Sonchus* plants showed higher concentrations of other notable bioactive compounds with health-promoting properties, such as *β*-carotene and vitamin C, compared to harvested plants.

## 3. Materials and Methods

The two-year study included both wild and cultivated harvesting.

### 3.1. Wild Plant Gathering

**Figure 4:**
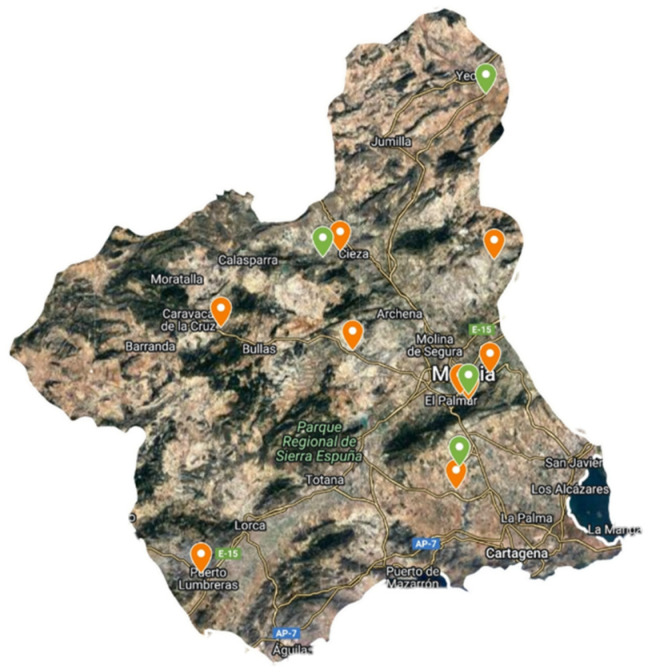
Location of *S. oleraceus* (orange) and *S. tenerrimus* (green) samples within the Region of Murcia.

**Figure 5:**
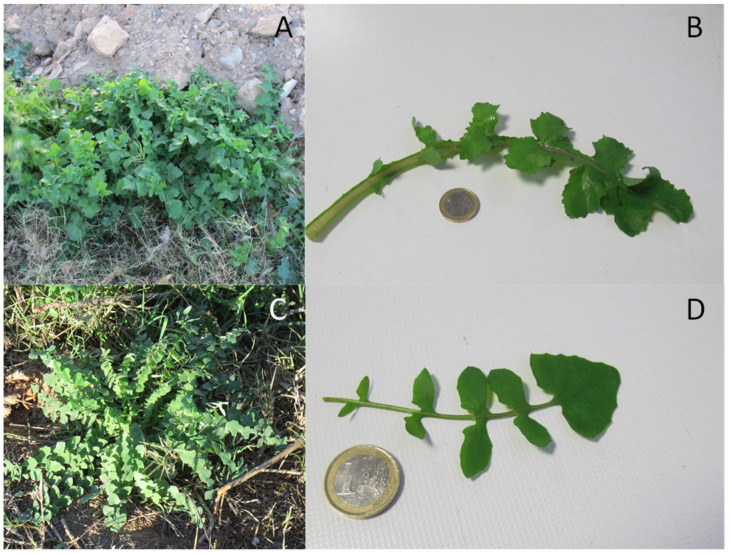
Plant and leaf detail of *S. oleraceus* (**A**,**B**) and *S. tenerrimus* (**C**,**D**).

In the first year, species were collected from the wild at different sites, mainly in the Region of Murcia (Figure 4). Plants were identified by Antonio Robledo Miras (Bsc in Botany). The collection protocol was the standard for this type of collection, recording data on date, collector, locality, coordinates, altitude, habitat, land use, geology, drainage, number of sampled plants and population size. Figure 5 shows images of plants and leaf details of *S. oleraceus* and *S. tenerrimus*. To obtain representative data, five and twelve batches of *S. oleraceus* and *S. tenerrimus* plants, respectively, were collected in different locations, and each batch was considered an independent sample, with at least 500 g of the edible portion of the plant. In addition, whole alive plants were collected and grown at IMIDA’s facilities to obtain seeds for further germination and cultivation trials, as well as for storage in the conservation collection of IMIDA’s germplasm bank (BAGERIM). Seeds were manually cleaned without applying any disinfection process in order not to alter their natural germination.

### 3.2. Germination Assay in Petri Dishes

For each species, one hundred seeds were placed in three Petri dishes (33, 33 and 34 seeds per dish) with filter paper. Water was added on demand to maintain humidity. Seeds were placed in a controlled environment growth chamber with a 16/8 h light/night photoperiod, at a 25 °C (day)/20 °C (night) temperature, 75% relative humidity and 350 μmol m^−2^ s^−1^ flux density. Seeds were considered germinated when the radicle appeared and were removed from the plate. The test lasted 15 days.

### 3.3. Field Germination Assays

In spring, a direct seeding trial was carried out in the experimental field of IMIDA in La Alberca (Murcia). Before sowing, the plot was tilled and compost was added in an amount of 1 kg m^−2^; this compost was incorporated into the soil by another pass of the tractor. Optimum plant density per surface area was estimated to be 400 plants m^−2^ and, to calculate the sowing doses for each species, germination data from the germination assay in Petri dishes and seed weight (seeds per gram) were taken into account. The unit plot had an area of 1 m^2^, and four replications for each species were established in randomized blocks. Sowing was carried out manually, and seeds were homogeneously distributed in each plot. Plots were then irrigated by means of an exudative pipe, and these irrigations continued until the end of the trial. Germination monitoring was carried out twenty-four days after sowing using a 60 cm × 60 cm template, placed in the centre of the 1 m^2^-plot, in which five 10 cm × 10 cm cells had been randomly chosen before.

### 3.4. Cultivation Trial

This experiment was conducted during the autumn–winter season. In order to achieve the maximum homogeneity in the cultivation trial, seed germination was initially carried out in Petri dishes, and after radicle emergence, germinated seeds were transferred to seedbeds. A standard commercial substrate for seedbeds was used, and trays were placed in a culture chamber with a photoperiod of 16 h of light and 8 h of darkness and a temperature range between 20 °C at night and 25 °C during the day; the humidity in the chamber was 75%. Subsequently, the seedlings were transplanted to the test plots with a planting density of 400 plants m^−2^ (planting frame 5 cm × 5 cm) following a randomized block distribution, with four blocks per species. Plants were sampled before flowering, when leaf length was approximately 5–10 cm, considering only the plant material from the youngest aerial part. Four replicates per species were collected, each replicate consisting of ten plants. Each replicate was pooled and, later in the same day, analysed for sensory attributes. Another portion was frozen in liquid nitrogen and left at −80 °C until metabolite analyses were carried out.

### 3.5. Metabolite Analysis

Metabolite extraction. An extraction from frozen material (3 g) with ultrapure water (10 mL) (Millipore, Molsheim, France) and ethyl acetate (25 mL) (J.T.Baker, Deventer, Holland) to eliminate pigment was carried out according to López et al. [33] to analyse soluble sugars, organic acids and total phenolic content. Carotenoids and chlorophylls were extracted from frozen material (1 g) according to Hernández et al. [56] with methanol/tetrahydrofuran (1:1, *v*/*v*) (25 mL) (Scharlau, Sentmenat, Spain) containing MgO (Merck, Darmstadt, Germany) and 0.1% (*w*/*v*) BHT (Sigma-Aldrich, Saint Louis, MO, USA); the final volume was 2 mL after evaporation. *β*-apo-8′-carotenal (Sigma-Aldrich, Saint Louis, MO, USA) was added as an internal standard. Vitamin C was extracted from 3 g of fresh material according to Fenoll et al. [73] with 0.05% (*w*/*v*) EDTA (Sigma-Aldrich, St. Louis, MO, USA) in water (10 mL).

Determination of sugar and organic acids. For soluble sugars, a Hewlett-Packard mod. 1100 (Waldbronn, Germany) with a refraction index (IR) detector was used. The column used for separation was a 300 × 7.8 mm i.d., CARBOSep CHO-682 LEAD column (Transgenomic, Omaha, NE, USA). The mobile phase was ultrapure water at a 0.4 mL·min^−1^ flow rate. To obtain the linearity of the detector response and the detection limits of sucrose, glucose and fructose (Sigma, Steinheim, Germany), standard solutions of those sugars (99.5% purity) were injected at concentrations of 1–10 g·L^−1^. Organic acids were analysed using liquid chromatography (Agilent 1200; Agilent Technologies, Santa Clara, CA, USA) equipped with a triple quadrupole mass spectrometer detector tandem-mass spectrometry (MS/MS) according to Flores et al. [74]. To obtain the linearity of the detector response, standard solutions were prepared using commercially available external standards from Sigma-Aldrich (St. Louis, MO, USA).

Chlorophyll and carotenoid analysis. Carotenoids and chlorophylls were determined by an Agilent HPLC (Agilent 1200; Agilent Technologies, Santa Clara, CA, USA) with a photodiode array detector (DAD) according to Hernández et al. [56] using methanol (solvent A) and methyl tert-butyl ether (solvent B) (Scharlau, Sentmenat, Spain) at a flow rate of 1.0 mL/min (15% solvent B for 20 min, a 20 min linear gradient to 30% solvent B, then maintained for 10 min and finally an 80 min linear gradient to 90% solvent B). Carotenoids and chlorophylls were quantified using commercially available external standards (DHI LAB, Hoersholm, Denmark). Luteoxanthin was quantified with respect to antheraxanthin. The *cis* isomers of *β*-carotene were quantified with respect to all-trans-*β*-carotene. 

Ascorbic acid analysis. Vitamin C (ascorbic and dehydroascorbic) was measured according to Fenoll et al. [73] using HPLC equipped with an MS/MS detector. Mobile phase was 0.2% (*v*/*v*) formic acid (Scharlau, Sentmenat, Spain) at a flow rate of 0.4 mL/min. Standard solutions of L-ascorbic (Sigma-Aldrich, St. Louis, MO, USA) were prepared with 0.05% (*w*/*v*) EDTA.

*Total phenolic content.* The Folin–Ciocalteu method was used to determine the total phenolic content. The standard curve consisted of gallic acid (Fluka, Steinheim, Germany) in concentrations ranging from 50 to 400 mg L^−1^. To calculate phenolic concentration, the optical density of each sample at 765 nm was measured in a Shimadzu UV-2401PC spectrophotometer (Kyoto, Japan).

### 3.6. Sensory Attributes

Sensory profiles of cultivated plants were determined with the application of a descriptive analysis by a panel of fifteen trained assessors. Plants were thoroughly washed and then submerged in water with food-grade hypochlorite. Finally, leaves were washed with abundant drinking water, and the excess water was removed with a kitchen centrifuge. They were refrigerated once again until the test was performed. Samples were presented raw, without any dressing, in food-grade plastic cups, and appropriately coded. The order in which the panellists tasted the samples was varied, and a random distribution was chosen. For the assessment of the colour and shape of the leaves, a separate sample was prepared with one leaf of each species. Samples were coded using a three-digit random number to avoid the possible influence that one or two numbers in sequential order or letters could have on the panellists’ judgments.

Tasters expressed their opinion preferably in numerical form for each of the studied variables, on the basis of an ideal pattern, according to a scale, or by means of answers to specific questions [75]. Firstly, the trained tasters judged five sensory attributes in a quantitative manner: colour, shape, smell, taste (flavour) and texture. To value each attribute, the nine-tier scale ranged from “I like it very much” (“9”, maximum value) to “I dislike it very much” (“1”, minimum value). Finally, panellists were asked about the degree of general acceptability on the basis of the above characteristics. A second questionnaire was carried out on taste characteristics (bitterness, sweetness, pungency [hotness], acidity and texture [crispness, stringiness or fibrousness]), on a scale of ten values, ranging from “0” (minimum value) to “9” (maximum value). Tasters had a space at the end of the questionnaire to write down their comments and include any relevant specific observations. The tasting took place between 12:30 p.m. and 14:00 p.m., in a quiet, bright place, where there was no smell to interfere with the process. Tasters carried out the tastings alone, and were given as much time as they deemed necessary. For evaluation, each assessor was provided with filtered water and asked to cleanse their palate between tastings.

### 3.7. Statistical Analysis

Data were subjected to analysis of variance (ANOVA) or Kruskal–Wallis’ test using the IBM SPSS Advanced Statistics 25.

## 4. Conclusions

The results of the composition and organoleptic and sensory properties of *S. oleraceus* and *S. tenerrimus* support the idea of their potential to be used as edible leafy vegetables and as promising assets for functional foods. They have proven to be a good source of bioactive compounds such as vitamins and pro-vitamins (vitamin C and *β*-carotene), carotenoids with recognized health-promoting properties (lutein, violaxanthin, neoxanthin and luteoxanthin) and compounds with well-known antioxidant properties (phenolic compounds and chlorophylls). The sensory profile demonstrated a good general acceptance of both species, indicating that they could be included in the diet. The cultivation of wild species is often associated with a loss of functional quality, but comparative results of wild versus cultivated plants often show very different results due to the great variability of habitats in which wild plants tend to grow spontaneously and different agricultural management techniques are carried out for cultivated plants. Our study is representative of spontaneously grown and cultivated *Sonchus* plants in a semi-arid Mediterranean climate. Although the results showed that the cultivation of *S. oleraceus* could cause a 57% decrease in the concentration of phenolic compounds, the opposite occurred with vitamin C (93% increase). In the case of *S. tenerrimus*, cultivation increased the content of sugars (98%) and organic acids (73%) and other compounds with important nutritional and health properties, such as *β*-carotene (31%) and vitamin C (58%). Therefore, the cultivation of these wild plants for commercialization would allow for a continuous and uniform supply of plants, without a significant decrease in quality. These aspects are of great relevance to ensure the viability of these crops in the agricultural systems of rural areas of the Mediterranean, avoiding the depletion of natural areas. Although more studies are needed to fully understand their cultivation potential, culinary applications and specific health benefits, the consumption of these species is a promising avenue to diversify food sources and promote sustainable nutrition.

## Figures and Tables

**Table 1:** Primary and secondary metabolite concentrations (µg g_FW_^−1^) in cultivated *S. oleraceus* L. and *S. tenerrimus* L. Values are mean ± SD.

Metabolite	*S. oleraceus* L.	*S. tenerrimus* L.	Sig.
Glucose	2414.8 ± 309.5	2406.4 ± 129.5	n.s.
Sucrose	748.7 ± 558.8	2743.2 ± 61.1	*
Fructose	524.1 ± 62.7	707.8 ± 79.2	*
Citric	875.6 ± 26.0	893.1 ± 214.7	n.s.
Malic	774.5 ± 30.3	1343.8 ± 280.4	*
Tartaric	356.0 ± 14.3	205.4 ± 70.1	**
Fumaric	71.6 ± 9.5	42.7 ± 8.5	*
Succinic	65.5 ± 2.8	67.7 ± 21.4	n.s.
Quinic	29.5 ± 2.4	38.0 ± 9.5	n.s.
Malonic	24.0 ± 2.3	10.7 ± 3.3	**
Isocitric	17.5 ± 0.2	20.0 ± 7.5	n.s.
Ketoglutaric	6.6 ± 0.6	21.8 ± 6.6	**
Glutamic	3.9 ± 0.2	5.5 ± 1.9	n.s.
Shikimic	3.0 ± 0.2	6.3 ± 0.9	**
Chlorophyll a	130.5 ± 41.7	147.4 ± 3.7	n.s.
Chlorophyll b	51.6 ± 16.4	56.2 ± 1.9	n.s.
*All*-*trans*-*β*-carotene	42.9 ± 6.5	49.8 ± 1.2	n.s.
Lutein	20.3 ± 3.2	18.9 ± 0.1	n.s.
*All*-*trans*-violaxanthin	9.6 ± 0.8	11.9 ± 1.3	n.s.
9 *cis*-Neoxanthin	4.9 ± 1.0	5.1 ± 0.5	n.s.
9 *cis*-*β*-carotene	4.5 ± 0.7	5.5 ± 0.3	n.s.
Luteoxanthin	3.8 ± 0.3	5.6 ± 2.1	n.s.
13 *cis*-*β*-carotene	1.8 ± 0.3	2.4 ± 0.7	n.s.
Vitamin C	527.7 ± 16.9	436.1 ± 4.4	**
Total phenolics	188.7 ± 9.2	328.6 ± 17.7	***

*, **, *** Significant differences between means at 5, 0.5 or 0.1% level of probability, respectively; n.s., non-significant at *p* = 5%.

## Data Availability

Data are available upon reasonable request from the authors.

## Supplementary Materials

The following supporting information can be downloaded at: https://www.mdpi.com/article/10.3390/plants13020269/s1, Table S1: Primary metabolites in wild-collected (year 1) *Sonchus* plants; Table S2: Secondary metabolites in wild-collected (year 1) *Sonchus* plants.

## References

[1] World Health Organization (2013). WHO Traditional Medicine Strategy 2014–2023.

[2] FAO (2002). Biodiversity and the Ecosystem Approach in Agriculture, Forestry and Fisheries. Proceedings of the Ninth Regular Session of the Commission on Genetic Resources for Food and Agriculture.

[3] Sánchez-Mata M.C., Tardio J. (2016). Mediterranean wild edible plants. Ethnobotany and Food Composition Tables.

[4] Romojaro A., Botella M.A., Obon C., Pretel M.T. (2013). Nutritional and antioxidant properties of wild edible plants and their use as potential ingredients in the modern diet. Int. J. Food Sci. Nutr..

[5] de Medeiros P.M., Figueiredo K.F., Santos Goncalves P.H., Caetano R.d.A., da Costa Santos E.M., Cota dos Santos G.M., Barbosa D.M., de Paula M., Mapeli A.M. (2021). Wild plants and the food-medicine continuum-an ethnobotanical survey in Chapada Diamantina (Northeastern Brazil). J. Ethnobiol. Ethnomed..

[6] Clemente-Villalba J., Burlo F., Hernandez F., Carbonell-Barrachina A.A. (2023). Valorization of wild edible plants as food ingredients and their economic value. Foods.

[7] Ajayi A.F., Akhigbe R.E., Adewumi O.M., Okeleji L.O., Mujaidu K.B., Olaleye S.B. (2012). Effect of ethanolic extract of *Cryptolepis sanguinolenta* stem on *in vivo* and *in vitro* glucose absorption and transport: Mechanism of its antidiabetic activity. Indian J. Endocrinol. Metab..

[8] Li W., Sun H., Zhou J., Zhang Y., Liu L., Gao Y. (2015). Antibacterial activities, antioxidant contents and antioxidant properties of three traditional Chinese medicinal extracts. Bangladesh J. Pharmacol..

[9] Huyan T., Li Q., Wang Y.L., Li J., Zhang J.Y., Liu Y.X., Shahid M.R., Yang H., Li H.Q. (2016). Anti-tumor effect of hot aqueous extracts from *Sonchus oleraceus* (L.) L. and *Juniperus sabina* L.—Two traditional medicinal plants in China. J. Ethnopharmacol..

[10] Łuczaj Ł., Pieroni A., Sánchez-Mata M.C., Tardío J. (2016). Nutritional Ethnobotany in Europe: From Emergency Foods to Healthy Folk Cuisines and Contemporary Foraging Trends. Mediterranean Wild Edible Plants.

[11] Zhu G., Mosyakin S.L., Clemants S.E. (1999). Flora of China.

[12] Fashir G.A., Abdalla N.I., and Fangama I.M. (2015). Assessment the consumption of *Sonchus cornutus* (Hochst) in Khartoum State, Sudan. Int. J. Curr. Microbiol. App. Sci..

[13] Seal T., Chaudhuri K., Pillai B. (2023). Nutritional and toxicological aspects of selected wild edible plants and significance for this society. S. Afr. J. Bot..

[14] Guil-Guerrero J.L., Giménez-Giménez A., Rodríguez-García I., Torija-Isasa M.E. (1998). Nutritional composition of *Sonchus* species (*S asper* L, *S oleraceus* L and *S tenerrimus* L). J. Sci. Food Agric..

[15] Li X.M., Yang P.L. (2018). Research progress of *Sonchus* species. Int. J. Food Prop..

[16] Khan R.A. (2012). Protective effects of *Sonchus asper* (L.) Hill, (*Asteraceae*) against CCl_4_-induced oxidative stress in the thyroid tissue of rats. BMC Complement. Altern. Med..

[17] de Paula Filho G.X., Barreira T.F., Pinheiro-Sant’Ana H.M. (2022). Chemical composition and nutritional value of three *Sonchus* species. Int. J. Food Sci..

[18] Li Q., Dong D.D., Huang Q.P., Li J., Du Y.Y., Li B., Li H.Q., Ting H. (2017). The anti-inflammatory effect of *Sonchus oleraceus* aqueous extract on lipopolysaccharide stimulated RAW 264.7 cells and mice. Pharm. Biol..

[19] Elhady S.S., Abdelhameed R.F.A., Mehanna E.T., Wahba A.S., Elfaky M.A., Koshak A.E., Noor A.O., Bogari H.A., Malatani R.T., Goda M.S. (2022). Metabolic profiling, chemical composition, antioxidant capacity, and *in vivo* hepato- and nephroprotective effects of *Sonchus cornutus* in mice exposed to cisplatin. Antioxidants.

[20] Vecchia C.A.D., Locateli G., Serpa P.Z., Gomes D.B., Ernetti J., Miorando D., Zanatta M.E.D.C., Silva Nunes R.K., Wildner S.M., Gutierrez M.V. (2022). *Sonchus oleraceus* L. promotes gastroprotection in rodents via antioxidant, anti-inflammatory, and antisecretory activities. Evid.-Based Complement. Altern. Med..

[21] Jimoh F.O., Adedapo A.A., Afolayan A.J. (2011). Comparison of the nutritive value, antioxidant and antibacterial activities of *Sonchus asper* and *Sonchus oleraceus*. Rec. Nat. Prod..

[22] Klos M., van de Venter M., Milne P.J., Traore H.N., Meyer D., Oosthuizen V. (2009). *In vitro* anti-HIV activity of five selected South African medicinal plant extracts. J. Ethnopharmacol..

[23] Jain S.K., Singh G.K. (2014). Preliminary phytochemical screening and *in vitro* antioxidant activity of extracts of whole plant of *Sonchus oleraceus* Asteraceae. Res. J. Pharm. Sci..

[24] Karar E.G.M. (2015). Phytochemical Characterization and Antimicrobial Activity of Sudanese Medicinal Plants. Doctoral Dissertation.

[25] Al Juhaimi F., Ghafoor K., Ahmed I.A.M., Babiker E.E., Ozcan M.M. (2017). Comparative study of mineral and oxidative status of *Sonchus oleraceus*, *Moringa oleifera* and *Moringa peregrina*. J. Food Meas. Charact..

[26] Cardoso Vilela F., Soncini R., Giusti-Paiva A. (2009). Anxiolytic-like effect of *Sonchus oleraceus* L. in mice. J. Ethnopharmacol..

[27] Ou Z.Q., Rades T., McDowell A. (2015). Anti-ageing effects of *Sonchus oleraceus* L. (puha) leaf extracts on H_2_O_2_-induced cell senescence. Molecules.

[28] Li X.Y., Liu Y.H., Wang B., Chen C.Y., Zhang H.M., Kang J.X. (2018). Identification of a sustainable two-plant diet that effectively prevents age-related metabolic syndrome and extends lifespan in aged mice. J. Nutr. Biochem..

[29] Tardio J., Pardo-De-Santayana M., Morales R. (2006). Ethnobotanical review of wild edible plants in Spain. Bot. J. Linn. Soc..

[30] Lentini F., Venza F. (2007). Wild food plants of popular use in Sicily. J. Ethnobiol. Ethnomed..

[31] Ceccanti C., Landi M., Benvenuti S., Pardossi A., Guidi L. (2018). Mediterranean wild edible plants: Weeds or “New functional crops”?. Molecules.

[32] Flores P., López A., Fenoll J., Hellín P., Kelly S. (2013). Classification of organic and conventional sweet peppers and lettuce using a combination of isotopic and bio-markers with multivariate analysis. J. Food Compos. Anal..

[33] López A., Javier G.A., Fenoll J., Hellín P., Flores P. (2014). Chemical composition and antioxidant capacity of lettuce: Comparative study of regular-sized (Romaine) and baby-sized (Little Gem and Mini Romaine) types. J. Food Compos. Anal..

[34] Schmitzer V., Senica M., Slatnar A., Stampar F., Jakopic J. (2021). Changes in metabolite patterns during refrigerated storage of lamb’s lettuce *Valerianella locusta* L. Betcke). Front. Nutr..

[35] Spinardi A., Ferrante A. (2012). Effect of storage temperature on quality changes of minimally processed baby lettuce. J. Food Agric. Environ..

[36] Fabian F.W., Blum H.B. (2006). Relative taste potency of some basic food constituents and their competitive and compensatory action. J. Food Sci..

[37] Schifferstein H.N.J., Frijters J.E.R. (1990). Sensory integration in citric-acid sucrose mixtures. Chem. Senses.

[38] Bonnans S., Noble A.C. (1993). Effect of sweetener type and of sweetener and acid levels on temporal perception of sweetness, sourness and fruitiness. Chem. Senses.

[39] Pangborn R.M. (1963). Relative taste intensities of selected sugars and organic acids. J. Food Sci..

[40] Derrien M., Aghabararnejad M., Gosselin A., Desjardins Y., Angers P., Boumghar Y. (2018). Optimization of supercritical carbon dioxide extraction of lutein and chlorophyll from spinach by-products using response surface methodology. LWT-Food Sci. Technol..

[41] Znidarcic D., Ban D., Sircelj H. (2011). Carotenoid and chlorophyll composition of commonly consumed leafy vegetables in Mediterranean countries. Food Chem..

[42] Leite A.C., Ferreira A.M., Morais E.S., Khan I., Freire M.G., Coutinho J.A.P. (2018). Cloud point extraction of chlorophylls from spinach leaves using aqueous solutions of non-ionic surfactants. ACS Sustain. Chem. Eng..

[43] Pérez-Gálvez A., Viera I., Roca M. (2020). Carotenoids and chlorophylls as antioxidants. Antioxidants.

[44] Ferruzzi M.G., Böhm V., Courtney P.D., Schwartz S.J. (2002). Antioxidant and antimutagenic activity of dietary chlorophyll derivatives determined by radical scavenging and bacterial reverse mutagenesis assays. J. Food Sci..

[45] Sarkar S., Manna M.S., Bhowmick T.K., Gayen K. (2020). Extraction of chlorophylls and carotenoids from dry and wet biomass of isolated *Chlorella Thermophila*: Optimization of process parameters and modelling by artificial neural network. Process Biochem..

[46] Panfili G., Niro S., Bufano A., D’Agostino A., Fratianni A., Paura B., Falasca L., Cinquanta L. (2020). Bioactive compounds in wild Asteraceae edible plants consumed in the Mediterranean Diet. Plant Foods Hum. Nutr..

[47] Kandlakunta B., Rajendran A., Thingnganing L. (2008). Carotene content of some common (cereals, pulses, vegetables, spices and condiments) and unconventional sources of plant origin. Food Chem..

[48] Gayathri G.N., Platel K., Prakash J., Srinivasan K. (2004). Influence of antioxidant spices on the retention of β-carotene in vegetables during domestic cooking processes. Food Chem..

[49] Blaner W.S., Marriott B.P., Birt D.F., Stallings V.A., Yates A.A. (2020). Vitamin A and Provitamin A Carotenoids. Present Knowledge in Nutrition.

[50] Johra F.T., Bepari A.K., Bristy A.T., Reza H.M. (2020). A Mechanistic review of *β*-carotene, lutein, and zeaxanthin in eye health and disease. Antioxidants.

[51] Mercadante A.Z., Rodriguez-Amaya D.B. (1990). Carotenoid composition and vitamin A value of some native Brazilian green leafy vegetables. Int. J. Food Sci. Technol..

[52] Paradiso R., Di Mola I., Cozzolino E., Ottaiano L., El-Nakhel C., Rouphael Y., Mori M. (2023). Nutrient and nutraceutical quality of rocket as a function of greenhouse cover film, nitrogen dose and biostimulant application. Agronomy.

[53] Kimura M., Rodriguez-Amaya D.B. (2002). A scheme for obtaining standards and HPLC quantification of leafy vegetable carotenoids. Food Chem..

[54] Calvo M.M. (2005). Lutein: A valuable ingredient of fruit and vegetables. Crit. Rev. Food Sci. Nutr..

[55] Lakshminarayana R., Raju M., Krishnakantha T.P., Baskaran V. (2005). Determination of major carotenoids in a few Indian leafy vegetables by high-performance liquid chromatography. J. Agric. Food Chem..

[56] Hernández V., Botella M.A., Hellín P., Cava J., Fenoll J., Mestre T., Martínez V., Flores P. (2021). Phenolic and carotenoid profile of lamb’s lettuce and improvement of the bioactive content by preharvest conditions. Foods.

[57] Kopsell D.A., Kopsell D.E., Curran-Celentano J., Wenzel A.J. Genetic variability for lutein concentrations in leafy vegetable crops can influence serum carotenoid levels and macular pigment optical density in human subjects. Proceedings of the II International Symposium on Human Health Effects of Fruits and Vegetables: Favhealth 2007.

[58] Krinsky N.I., Landrum J.T., Bone R.A. (2003). Biologic mechanisms of the protective role of lutein and zeaxanthin in the eye. Annu. Rev. Nutr..

[59] Bartlett H.E., Ramawat K., Mérillon J.M. (2013). Xanthophylls and the eye. Natural Products.

[60] Demmig-Adams B., Lopez-Pozo M., Stewart J.J., Adams W.W. (2020). Zeaxanthin and Lutein: Photoprotectors, anti-Inflammatories, and brain food. Molecules.

[61] de Sá M.C., Rodriguez-Amaya D.B. (2003). Carotenoid composition of cooked green vegetables from restaurants. Food Chem..

[62] Rumengan A.P., Mandiangan E.S., Tanod W.A. (2021). Identification of pigment profiles and antioxidant activity of *Rhizophora mucronata* mangrove leaves origin Lembeh, North Sulawesi, Indonesia. Biodiversitas.

[63] Perry A., Rasmussen H., Johnson E.J. (2009). Xanthophyll (lutein, zeaxanthin) content in fruits, vegetables and corn and egg products. J. Food Compos. Anal..

[64] Molnar P., Deli J., Tanaka T., Kann Y., Tani S., Gyemant N., Molnar J., Kawase M. (2010). Carotenoids with anti-*Helicobacter pylori* activity from *Golden delicious* apple. Phytother. Res..

[65] Joshi B.C., Mukhija M., Kalia A.N. (2014). Pharmacognostical review of *Urtica dioica* L.. Int. J. Green Pharm..

[66] Upreti S., Prusty J.S., Pandey S.C., Kumar A., Samant M. (2021). Identification of novel inhibitors of angiotensin-converting enzyme 2 (ACE-2) receptor from *Urtica dioica* to combat coronavirus disease 2019 (COVID-19). Mol. Divers..

[67] Oh M.M., Carey E.E., Rajashekar C.B. (2011). Antioxidant phytochemicals in lettuce grown in high tunnels and open field. Hortic. Environ. Biotechnol..

[68] Gutiérrez-Velázquez M., Almaraz-Abarca N., Herrera-Arrieta Y., Ávila-Reyes J.A., González-Valdez L.S., Torres-Ricario R., Uribe-Soto J.N., Monreal-García H.M. (2018). Comparison of the phenolic contents and epigenetic and genetic variability of wild and cultivated watercress (*Rorippa nasturtium* var. aquaticum L.). Electron. J. Biotechnol..

[69] Ceccanti C., Landi M., Incrocci L., Pardossi A., Venturi F., Taglieri I., Ferroni G., Guidi L. (2020). Comparison of three domestications and wild-harvested plants for nutraceutical properties and sensory profiles in five wild edible herbs: Is domestication possible?. Foods.

[70] Riquelme J., Antonio Olaeta J., Galvez L., Undurraga P., Fuentealba C., Osses A., Orellana J., Gallardo J., Pedreschi R. (2016). Nutritional and functional characterization of wild and cultivated *Sarcocornia neei* grown in Chile. Cienc. Investig. Agrar..

[71] Paschoalinotto B.H., Polyzos N., Compocholi M., Rouphael Y., Alexopoulos A., Dias M.I., Barros L., Petropoulos S.A. (2023). Domestication of wild edible species: The response of *Scolymus hispanicus* plants to different fertigation regimes. Horticulturae.

[72] Kumar K., Debnath P., Sailendra Singh S., Kumar N. (2023). An overview of plant phenolics and their involvement in abiotic stress tolerance. Stresses.

[73] Fenoll J., Martínez A., Hellín P., Flores P. (2011). Simultaneous determination of ascorbic and dehydroascorbic acids in vegetables and fruits by liquid chromatography with tandem-mass spectrometry. Food Chem..

[74] Flores P., Hellín P., Fenoll J. (2012). Determination of organic acids in fruits and vegetables by liquid chromatography with tandem-mass spectrometry. Food Chem..

[75] Sancho J., Bota E., Castro J. (1999). Introducción al Análisis Sensorial de los Alimentos.

