# Supplementary material for: Chemical Composition of Wild Collected and Cultivated Edible Plants (Sonchus oleraceus L. and Sonchus tenerrimus L.)
Source: Plants (Basel). 2024 Jan 17;13(2):269. doi: 10.3390/plants13020269 (PMC10819898; doi:10.3390/plants13020269)
Supplement: Supplementary file 1 [file plants-13-00269-s001.zip › plants-2759337-supplementary.pdf]

plants

MDPI

Supplementary Table S1. Mean, minimum and maximum values of primary metabolite concentrations (μg g_{FW}^{-1}) in wild collected (year 1) *Sonchus* plants.

**Family**	**Metabolite**		***S. oleraceus***	***S. tenerrimus***
Soluble sugars	Glucose	Mean	1895.8	1691.8
		Min	587.8	176.0
		Max	3685.2	3862.1
	Fructose	Mean	1401.9	257.4
		Min	0.0	0.0
		Max	3631.1	1295.5
	Sucrose	Mean	2472.3	1110.5
		Min	405.7	159.8
		Max	5337.9	2590.6
Organic acids	Citric	Mean	116.1	106.4
		Min	40.6	7.2
		Max	322.5	421.6
	Malic	Mean	1410.3	842.0
		Min	157.7	144.6
		Max	2476.9	2526.1
	Tartaric	Mean	196.3	172.2
		Min	103.7	17.5
		Max	275.6	388.3
	Succinic	Mean	15.9	14.3
		Min	3.0	2.1
		Max	44.3	38.4
	Quinic	Mean	80.1	49.6
		Min	22.3	7.7
		Max	132.0	86.8
	Malonic	Mean	4.7	6.1
		Min	1.4	1.1
		Max	8.5	14.8
	Ketoglutaric	Mean	9.3	13.6
		Min	2.2	0.7
		Max	19.8	94.5
	Glutamic	Mean	19.5	17.5
		Min	13.7	1.6
		Max	27.6	36.1
	Shikimic	Mean	4.7	3.0
		Min	2.1	0.3
		Max	10.5	4.3

Supplementary Table 2. Mean, minimum and maximum values of secondary metabolite concentrations (µg g_{FW}^{-1}) in wild collected (year 1) *Sonchus* plants.

**Family**	**Metabolite**		***S. oleraceus***	***S. tenerrimus s***
Chlorophylls	Chlorophyll a	Mean	150.3	151.1
		Min	87.9	9.8
		Max	209.6	226.5
	Chlorophyll b	Mean	63.4	64.4
		Min	36.8	2.8
		Max	102.0	100.9
Carotenoids	*All-trans-β*-carotene	Mean	40.6	38.6
		Min	22.4	4.8
		Max	61.9	58.7
	Lutein	Mean	21.9	19.9
		Min	13.8	2.0
		Max	35.2	31.7
	All-trans-violaxanthin	Mean	6.2	4.9
		Min	4.3	0.9
		Max	10.3	7.9
	9 *cis*-Neoxanthin	Mean	8.0	7.3
		Min	3.8	1.5
		Max	13.9	16.1
	9 *cis-β*-carotene	Mean	4.2	4.0
		Min	2.4	0.5
		Max	6.1	6.1
	Luteoxanthin	Mean	8.6	7.9
		Min	2.4	0.9
		Max	20.3	14.3
	13 *cis-β*-carotene	Mean	1.7	1.5
		Min	0.9	0.2
		Max	2.7	2.5
Vitamins	Vitamin C	Mean	273.0	276.6
		Min	143.7	129.0
		Max	447.1	558.5
Phenolics	Total phenolics	Mean	0.4	0.3
		Min	0.1	0.2
		Max	0.9	0.7
